# Fundus albipunctatus: review of the literature and report of a novel *RDH5* gene mutation affecting the invariant tyrosine (p.Tyr175Phe)

**DOI:** 10.1007/s13353-015-0281-x

**Published:** 2015-03-28

**Authors:** Anna Skorczyk-Werner, Przemysław Pawłowski, Marta Michalczuk, Alicja Warowicka, Anna Wawrocka, Katarzyna Wicher, Alina Bakunowicz-Łazarczyk, Maciej R. Krawczyński

**Affiliations:** 1 Department of Medical Genetics, Poznan University of Medical Sciences, 8, Rokietnicka Street, 60-806 Poznań, Poland; 2 Department of Pediatric Ophthalmology and Strabismus, Medical University of Bialystok, 17, Waszyngtona Street, 15-275 Białystok, Poland; 3 Center for Medical Genetics “Genesis”, 4, Grudzieniec Street, 60-601 Poznań, Poland; 4 NanoBioMedical Centre, Adam Mickiewicz University, 85, Umultowska Street, 61-614 Poznań, Poland

**Keywords:** Fundus albipunctatus (FA), *RDH5* gene, Mutation, Invariant tyrosine, Short-chain dehydrogenases/reductases (SDRs), Retinal pigment epithelium (RPE)

## Abstract

Fundus albipunctatus (FA) is a rare, congenital form of night blindness with rod system impairment, characterised by the presence of numerous small, white-yellow retinal lesions. FA belongs to a heterogenous group of so-called flecked retina syndromes. This disorder shows autosomal recessive inheritance and is caused mostly by mutations in the *RDH5* gene. This gene encodes the enzyme that is a part of the visual cycle, the 11-*cis* retinol dehydrogenase. This study is a brief review of the literature on FA and a report of the first molecular evidence for *RDH5* gene mutation in a Polish patient with this rare disorder. We present a novel pathogenic *RDH5* gene mutation in a 16-year-old female patient with symptoms of night blindness. The patient underwent ophthalmological examinations, including colour vision testing, fundus photography, automated visual field testing, full-field electroretinography (ERG) and spectral optical coherent tomography (SOCT). The patient showed typical FA ERG records, the visual field was constricted and fundus examination revealed numerous characteristic, small, white-yellowish retinal lesions. DNA sequencing of the *RDH5* gene coding sequence (exons 2–5) enabled the detection of the homozygous missense substitution c.524A > T (p.Tyr175Phe) in exon 3. This is the first report of *RDH5* gene mutation that affects the invariant tyrosine, one of the most conserved amino acid residues in short-chain alcohol dehydrogenases/reductases (SDRs), crucial for these enzymes’ activity. The location of this substitution, together with its predicted influence on the protein function, indicate that the p.Tyr175Phe mutation is the cause of FA in our patient.

## Introduction

### Clinical characterisation of fundus albipunctatus

Fundus albipunctatus (FA; MIM 136880) is a rare, hereditary, in most cases stationary, retinal disease, which is characterised by impaired night vision and numerous small, white-yellowish retinal lesions placed throughout the retina, except the fovea (Sergouniotis et al. 2011). FA belongs to a heterogenous group of genetically determined flecked retina syndromes. The symptoms of these disorders include conditions characterised by multiple retinal yellowish-white lesions of various sizes and configuration, without vascular or optic nerve abnormalities. The group of flecked retina syndromes encompasses FA, retinitis punctata albescens, fundus flavimaculatus (Stargardt disease), familial drusen and fleck retina of Kandori, but far more diseases correspond to the rather vague definition of fleck retina syndromes (De Laey 1993; Walia et al. 2009). Moreover, there is a collection of diseases called white dots syndromes that can also be misdiagnosed with flecked retina syndromes. White dots syndromes are characterised by white lesions in the retinal pigment epithelium (RPE) or choroidal layers. The aetiology of these disorders is unknown, but these syndromes are suspected to be inflammatory in nature and can be associated with uveitis (Matsumoto et al. 2007).

FA is a form of congenital stationary night blindness. The symptoms of defective dark adaptation may not be perceptible to the affected person. The optic nerve heads and the retinal vessels show no signs of irregularity. The visual field and visual acuity examinations of patients suffering from FA do not detect any abnormalities unless a dim stimulus is used. Dim stimulus causes a worsening of visual acuity and a constriction of the visual field. The scotopic electroretinography (ERG) responses are reduced after a 30–40-min period of dark adaptation, but typically normalise after prolonged dark adaptation (Yamamoto et al. 1999; Sergouniotis et al. 2011; Wang et al. 2012). The photopic responses are usually normal if FA is not accompanied by macular dystrophy. Although long-term follow-up usually shows no progression in rods dysfunction in patients with this form of night blindness, some patients, especially the elderly, reveal progressive cone dystrophy (Nakamura et al. 2000, 2003; Wada et al. 2001; Niwa et al. 2005). Full-field photopic electroretinograms of these individuals are usually severely reduced, a bull’s eye maculopathy is often identified, and visual fields and acuity are impaired (Nakamura et al. 2000 2003). Recently, it has been estimated that cone dysfunction can affect more than 30 % of patients with FA (Niwa et al. 2005; Sergouniotis et al. 2011; Pras et al. 2012). Lidén and coworkers suggested that cone dystrophy may be either the result of impaired function of the RPE caused by a *RDH5* gene mutation or a direct consequence of a decreased supply of 11-*cis* retinal to the cones (Lidén et al. 2001).

### Genetic background

FA shows an autosomal recessive inheritance pattern. In one family with this retinal disease, autosomal dominant or pseudodominant inheritance was suggested (Kranias et al. 1981). FA is caused almost exclusively by mutations in the 11-*cis* retinol dehydrogenase 5 (*RDH5*) gene (Yamamoto et al. 1999). However, mutations in two other genes, retinaldehyde binding protein 1 (*RLBP1*) and RPE-specific protein (*RPE65*), are also known to be associated with FA (Naz et al. 2011; Schatz et al. 2011). Retinaldehyde binding protein 1 is expressed in the RPE and Müller cells of the neuroretina, where it carries 11-*cis* retinol and 11-*cis* retinaldehyde as ligands (Sparkes et al. 1992). Only a few *RLBP1* gene mutations in patients with FA have been reported to date (Katsanis et al. 2001; Naz et al. 2011). Katsanis end coworkers (2001) found a p.Arg150Gln mutation in the *RLBP1* gene in a consanguineous Saudi Arabian kindred with a retinal dystrophy phenotype that fulfilled the criteria of FA in younger individuals and retinitis punctata albescens in older patients. Two homozygous *RLBP1* gene mutations (p.Arg156* and p.Gly116Arg) have also been identified in two unrelated, consanguineous Pakistani families suffering from FA (Naz et al. 2011). RPE-specific protein (*RPE65*) is the isomerase of the visual cycle, catalysing the conversion of all-*trans* retinyl ester to 11-*cis* retinol (Moiseyev et al. 2005). To date, mutations in the *RPE65* gene associated with FA have only been reported in one paper. A compound heterozygote of IVS + 5G > A and c.344 T > C mutations in the *RPE65* gene was found in a patient with FA (Schatz et al. 2011).

Most cases of FA are caused by mutations in the *RDH5* gene (Gonzalez-Fernandez et al. 1999). The *RDH5* gene encodes the enzyme that is a part of the visual cycle, the 11-*cis* retinol dehydrogenase (Simon et al. 1995). The retinoid (visual) cycle is an enzyme pathway that occurs to regenerate the visual chromophore following light exposure (Travis et al. 2007). 11-*cis* retinol dehydrogenase (*RDH5*) is predominantly expressed in the smooth endoplasmic reticulum of the RPE of the eye (Simon et al. 1996). RPE cells play multiple roles essential for visual function, such as involvement in the uptake and metabolic processing of retinoids in the visual cycle (Simon et al. 1999). *RDH5* has an important role in the molecular background of vision, as it catalyses the final step in the biosynthesis of 11-*cis* retinaldehyde, the universal chromophore of visual pigments (Simon et al. 1995). Absorption of a photon by an opsin pigment causes isomerisation of the chromophore from 11-*cis* retinaldehyde to all-*trans* retinaldehyde. After entering the RPE cell, all-*trans* retinol is transferred into all-*trans* retinyl esters, which are isomerised by *RPE65* (RPE-specific) protein into 11-*cis* retinol esters (11-*cis* retinol). Then, 11-*cis* retinol is transported through the subretinal space, where it is oxidated by the *RDH5* enzyme into 11-*cis* retinal (Simon et al. 1995; Wang et al. 2012).

**Fig. 1:**
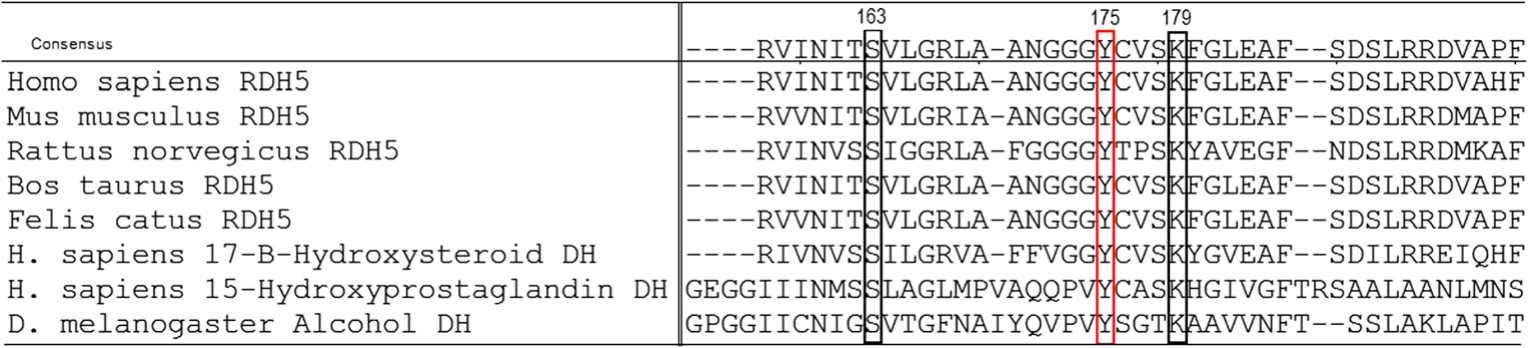
The amino acid conservation of part of the active site (residues 157–196 according to the numbering system of the *RDH5* protein) among *RDH5* of several species and three other short-chain dehydrogenases. The red frame indicates the invariant tyrosine, while the black frame indicates two other highly conserved residues involved in the catalytic mechanism: serine-163 and lysine-179. The abbreviation ‘DH’ in the names of three aligned proteins’ sequences stands for ‘dehydrogenase’

Retinol dehydrogenase 5 protein consists of 318 amino acids and is a member of the short-chain dehydrogenases/reductases (SDR) superfamily (Simon et al. 1996). This family encompasses at least 57 varied, well-characterised enzymes (Jörnvall et al. 1995), which catalyse the metabolism of steroids, fatty acids, carbohydrates, amino acids and aromatic compounds (Marchler-Bauer et al. 2015). Although the amino acids sequence identity between the members of this large protein family is only at the 15–30 % level, there are two well-conserved regions within the enzymes’ sequences: the motif Gly-X-X-X-Gly-X-Gly, consisting of three glycines within a cofactor binding site for NAD(H) or NADP(H), and the amino acid motif Tyr-X-X-X-Lys, with an invariant tyrosine residue inside the active site. Like all the classical SDR enzymes, the *RDH5* amino acid sequence contains these two conserved domains: the motif Gly-Cys-Asp-Ser-Gly-Phe-Gly at the amino acid residues 35–41 and the sequence encompassing the invariant tyrosine Tyr-Cys-Val-Ser-Lys at the residues 175–179 (Persson et al. 1991; Jörnvall et al. 1995; Simon et al. 1999). Retinol dehydrogenase 5 protein is highly conserved among species (Simon et al. 1996). The amino acid conservation of part of the active site (encompassing three conserved residues: Ser-163, Tyr-175, Lys-179) among *RDH5* of several species and three other short-chain dehydrogenases is shown in Fig. 1. *RDH5* is an integral membrane protein (Simon et al. 1995). It is composed of the N-terminus (18 amino acids) located within the membrane, the ectodomain encompassing the active site, which is present in the lumen of the smooth endoplasmic reticulum (SER) (residues 19–288), the C-terminal membrane-spanning domain (289–310 amino acids) and the C-terminal tail (311–318 amino acids) located in the cytosol of the RPE (Simon et al. 1999; Ajmal et al. 2012). The different localisation of the *RDH5* domains within the RPE cell suggests that biosynthesis of 11-*cis* retinaldehyde is a compartmentalised process (Simon et al. 1999).

The *RDH5* gene is mapped on the chromosome 12q13-q14. The transcript (ENST00000257895) spans 1,269 bp and contains five exons, including four coding exons (2–5). The lengths of the coding exons are as follows: exon 2–342 bp (32 bp of the 5′ untranslated region and 310 bp translated sequence), exon 3–259, exon 4–164 and exon 5–384 bp (the translated part is 221 bp) (Simon et al. 1996).

## Materials and methods

This study was conducted in accordance with the tenets of the Declaration of Helsinki. A 16-year-old female patient of Polish origin with clinical signs of night blindness was examined. The patient underwent colour vision testing, fundus photography, automated visual field testing (Humphrey–Zeiss), full-field ERG and spectral optical coherent tomography (SOCT). The electrophysiological examinations included a full-field ERG protocol with the standard scotopic 20 min dark adaptation and extended protocol with prolonged 120 min of dark adaptation. The standard photopic ERG (30 Hz white flicker stimulation) was performed after 10 min of light adaptation.

Voluntary informed consent for genetic examination was obtained not only from the mother of the patient (as the patient was underaged), but also from both parents and two sisters, who had their blood taken for segregation analysis for the presence of the novel mutation. Genomic DNA was extracted from peripheral blood using the conventional salting-out procedure. The coding regions of the *RDH5* gene (exons 2–5) were amplified and sequenced to screen for disease-causing mutations in the patient. Eight primer pairs (including three pairs for amplifying exon 2, two for exons 3 and 5, and one pair for exon 4) were used following a previous report (Yamamoto et al. 1999). A fragment of exon 3 (primer pair designed 3b) was also amplified in both parents and two sisters of the proband. The polymerase chain reaction (PCR) products were purified with the use of ExoSAP-IT (Exonuclease I and Shrimp Alkaline Phosphatase Cleanup for PCR products, Affymetrix) and directly sequenced using Dye Terminator chemistry (v3.1 BigDye® Terminator, Life Technologies). The sequencing products were separated on an ABI 3130xl capillary sequencer (Applied Biosystems). The obtained sequences were verified by comparing them to the reference sequence of the *RDH5* gene (GenBank NM_001199771.1) and screened for mutations. The in silico analysis using PROVEAN (Choi et al. 2012), SIFT (Kumar et al. 2009) and PolyPhen-2 (Adzhubei et al. 2010) software was performed to assess the possible functional effect of the novel missense mutation.

## Results

### Family history

**Fig. 2:**
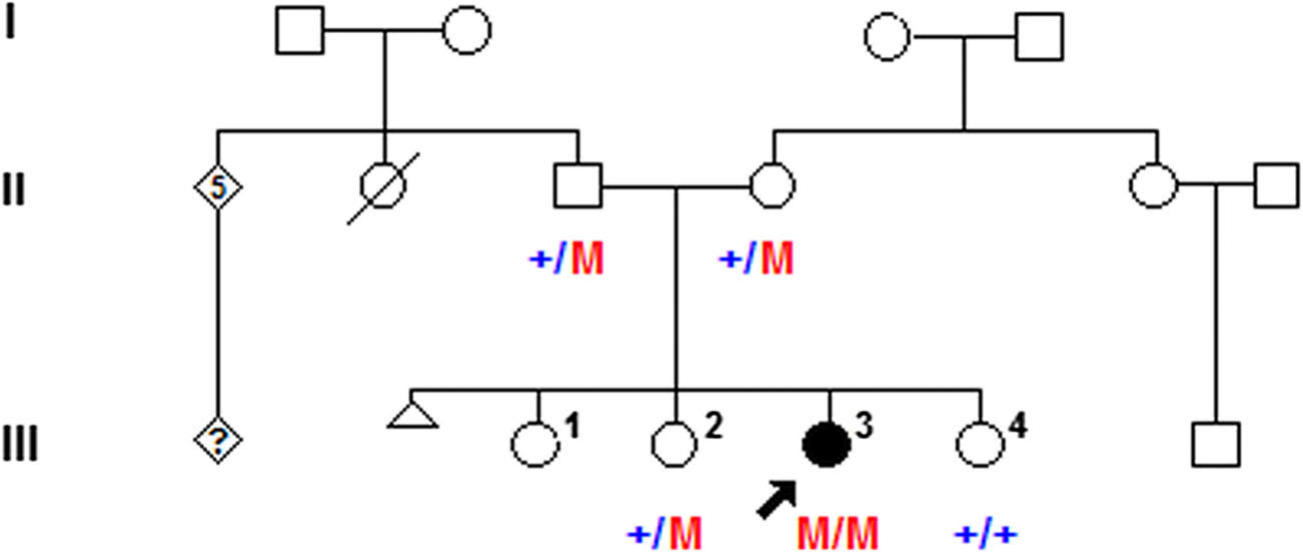
Pedigree and genotypes at the *RDH5* gene nucleotide position 524 of the family with the c.524A > T mutation. The mutation is marked with red ‘M’ letter, while the blue ‘+’ symbol indicates a wild-type allele. The parents and two sisters of the proband were involved in the exon 3 sequencing analysis

The patient has three sisters. The younger sister suffers from astigmatism, while the mother and the two older sisters had no ophthalmologic problems. In both the proband’s father’s eyes, presenile cataract was revealed at the age of 38 years. He suffered from retinal detachment in the right eye later on. The first pregnancy of the patient’s mother ended with a stillbirth (pedigree, Fig. 2).

### Clinical status

**Fig. 3:**
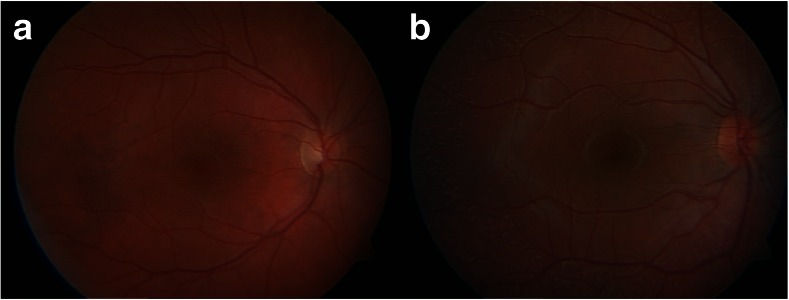
Fundus examination. **a** The right eye of a healthy individual, **b** The right eye of the patient with the c.524A > T mutation in the *RDH5* gene. Numerous small, white-yellowish retinal lesions are located in the upper segments of the retina

**Fig. 4:**
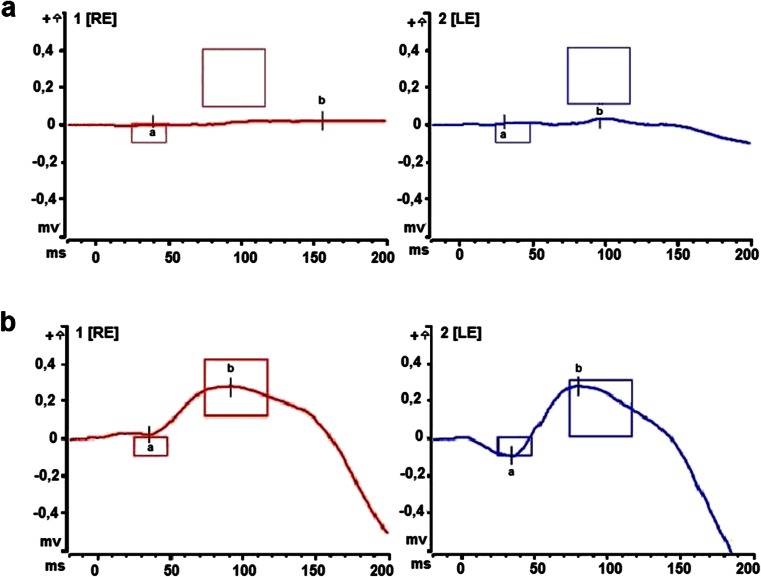
Comparison of scotopic responses after 30 and 120 min of dark adaptation: *RE*, right eye; *LE*, left eye. **a** The reduction of scotopic responses (DA 0.009 cdxs/m^2^) (b-wave amplitude RE: 26.68 μV, LE: 26.53 μV, normal 260 ± 151.4 μV), standard electroretinography (ERG) response (3 cdxs/m^2^) on the borderline after 30 min of dark adaptation. **b** The normalisation of scotopic responses after 120 min of scotopic adaptation (b-wave amplitude RE: 259.1 μV, LE: 378.2 μV)

Visual acuity and colour vision in the patient were normal. The examination of the anterior segment and the pupillary reflexes showed no abnormalities. An eye fundus examination revealed numerous small white-yellowish retinal lesions mainly in the upper quadrants of the retina (Fig. 3). Examination of the visual field revealed its peripheral constriction to approximately 10–20°. A full-field electroretinogram showed significantly reduced scotopic responses after the standard period of 20–30 min dark adaptation (Fig. 4a). However, after a prolonged 120 min of dark adaptation, rod responses normalised (Fig. 4b). Photopic responses in all examinations were normal. The high-definition SOCT showed no abnormalities of the central macular thickness in either eye, but local modulations of the RPE and IS/OS (inner segment–outer segment) junctions corresponding with retinal flecks were identified.

### Genetic analysis

**Fig. 5:**
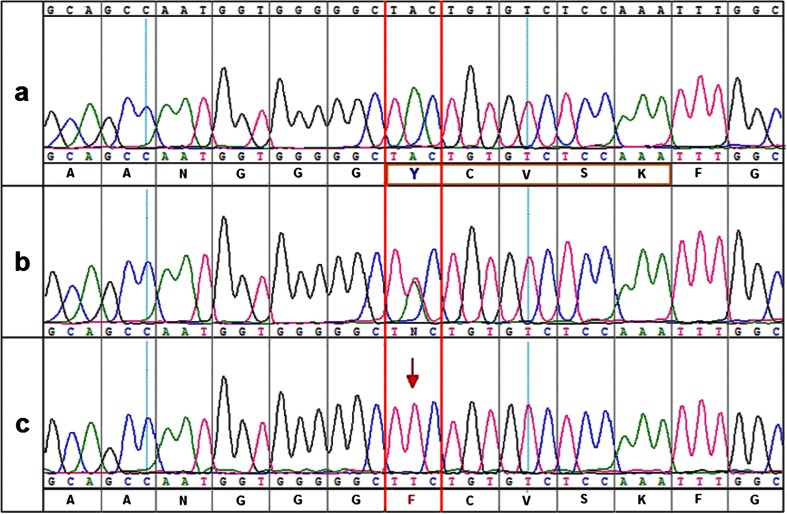
A chromatogram showing the c.524A > T mutation in the *RDH5* gene. **a** The wild-type nucleotide sequence and the wild-type protein sequence. The orange frame indicates the most conservative element between short-chain alcohol dehydrogenases, located within the active site of the enzyme. Invariant tyrosine is labelled blue and indicated with the red frame. **b** The nucleotide sequence of the heterozygous parent. **c** The sequence of the patient with c.524A > T mutation and truncated protein sequence

As the clinical findings, especially characteristic eye fundus appearance, indicated a suspicion of FA, searching for a mutation in the *RDH5* gene seemed to be the most appropriate strategy. Bidirectional sequencing of the *RDH5* gene coding region (exons 2–5) revealed a homozygous mutation c.524A > T in exon 3. This transversion changes codon UAC to UUC, which results in the substitution of polar tyrosine to non-polar phenylalanine at amino acid position 175 (p.Tyr175Phe) (Fig. 5). The in silico analysis of the predicted influence of the p.Tyr175Phe substitution on protein function with the use of PROVEAN software (the tolerance index score was −3.900), as well as SIFT software (tolerance index score 0.00), revealed that this amino acid change is deleterious. The in silico analysis using PolyPhen-2 software predicted the mutation to be probably damaging (score of 1). The c.524A > T variant was not found in a control cohort annotated in the Exome Variant Server (EVS) database (Exome Variant Server 2015) nor in the 1000 Genomes Project database (1000 Genomes Project Consortium 2012). The segregation analysis of the mutation in the proband’s family revealed that both parents and one of the proband’s sisters are heterozygous carriers of the c.524A > T substitution (pedigree, Fig. 2).

### Mutations in the *RDH5* gene: review of the literature

Since 1999, when Yamamoto et al. described the mutations in the *RDH5* gene in two unrelated patients suffering from FA (Yamamoto et al. 1999), there have been reports of missense, in-frame and frameshift mutations (Sergouniotis et al. 2011). To date, more than 40 mutations in the *RDH5* gene have been reported, and most of them are missense variants (Nakamura et al. 2000; Driessen et al. 2001; Sergouniotis et al. 2011; Ajmal et al. 2012; Wang et al. 2012; Waldron and Medefindt 2014; Stenson et al. 2014). The list of *RDH5* gene mutations identified in patients with FA together with the regions of the protein affected by these changes is shown in Table 1. The *RDH5* gene mutations have been identified mostly in homozygotic or compound heterozygotic forms, but a few variants (p.Arg19Gly, p.Arg191Gln and p.Arg278Gln) have been found as single heterozygous mutations (Sergouniotis et al. 2011; Pras et al. 2012). There seems to be no hot spot in the gene, as the reported mutations are distributed across the entire *RDH5* coding sequence. The mutations identified affect the entire protein, except the N-terminus, with most mutations located in the longest domain: lumenal ectodomain (see Table 1). The analyses of biochemical defects in *RDH5* mutants associated with FA revealed that all the mutations tested (marked with ‘+’ in Table 1) affect the stability and expression level of the protein and result in subcellular mislocalisation. Moreover, loss of enzymatic activity in vitro and in vivo has been observed for almost all the constructed mutants (except the mutant with amino acid change p.Ala294Pro located in the C-terminal domain) (Yamamoto et al. 1999; Cideciyan et al. 2000; Lidén et al. 2001). Even in the absence of the enzyme activity caused by many *RDH5* mutations, night vision regenerates after prolonged dark adaptation in patients with FA. This fact can be explained by the results of the studies on Rdh knockout mice models (Driessen et al. 2000; Kim et al. 2005). Driessen and coworkers revealed that transgenic mice missing the *RDH5* gene display delayed dark adaptation, but only at a very high bleach level. The studies on Rdh5 and Rdh11 knockout mice revealed that one more enzyme, RDH11, appeared to have an important role in regenerating the chromophore. These results indicate that both RDH5 and RDH11 contribute to 11-*cis* retinal production (Driessen et al. 2000).

**Table 1:** *RDH5* mutations identified in patients with fundus albipunctatus (FA)

Exon/intron	Nucleotide position	Amino acid residue	Region of the protein	Mutants analysis^a^	Reference
Exon 2	c.55A > G	p.Arg19Gly	Ectodomain	−	Sergouniotis et al. (2011)
Exon 2	c.71_74delTGCC	p.Leu24Profs*36	Ectodomain	−	Pras et al. (2012)
Exon 2	c.95delT	p.Phe32Serfs*29	Ectodomain	−	Schatz et al. (2010)
Exon 2	c.98 T > C	p.Ile33Thr	Ectodomain	−	Sergouniotis et al. (2011)
Exon 2	c.98 T > A	p.Ile33Asn	Ectodomain	−	Rüther et al. (2004)
Exon 2	c.103G > A	p.Gly35Ser	Ectodomain, the conserved cofactor binding motif	+	Nakamura et al. (2000); Wada et al. (2001)
Exon 2	c.124C > T	p.Arg42Cys	Ectodomain	−	Niwa et al. (2005)
Exon 2	c.129delT	p.Leu44Trpfs*17	Ectodomain	−	Driessen et al. (2001)
Exon 2	c.160C > T	p.Arg54*	Ectodomain	−	Pras et al. (2012)
Exon 2	c.175 T > A	p.Cys59Ser	Ectodomain	−	Wang et al. (2012)
Exon 2	c.214insGTGG	p.Val71fs*86	Ectodomain	−	Driessen et al. (2001)
Exon 2	c.218C > T	p.Ser73Phe	Ectodomain	+	Yamamoto et al. (1999)
Exon 2	c.285G > A	p.Trp95*	Ectodomain	−	Wang et al. (2012)
Intron 2	c.310 + 1G > A	–	Ectodomain	−	Sergouniotis et al. (2011)
Exon 3	c.319G > C	p.Gly107Arg	Ectodomain	−	Nakamura et al. (2000); Sato et al. (2004); Hotta et al. (2003)
Exon 3	c.346G > C	p.Gly116Arg	Ectodomain	−	Sergouniotis et al. (2011)
Exon 3	c.346_347insGCA	p.Gly116_Ile117insSer	Ectodomain	−	Sergouniotis et al. (2011)
Exon 3	c.382G > A	p.Asp128Asn	Ectodomain	+	Iannaccone et al. (2007); Schatz et al. (2010); Pras et al. (2012)
Exon 3	c.394 G > A	p.Val132Met	Ectodomain	−	Nakamura et al. (2000)
Exon 3	c.416G > T	p.Gly139Val	Ectodomain	−	Sergouniotis et al. (2011)
Exon 3	c.469C > T	p.Arg157Trp	Ectodomain	+	Cideciyan et al. (2000)
Exon 3	c.470G > A	p.Arg157Gln	Ectodomain	−	Hajali et al. (2009); Sergouniotis et al. (2011)
Exon 3	c.490G > T	p.Val164Phe	Ectodomain	−	Yamamoto et al. (2003)
Exon 3	c.500G > A	p.Arg167His	Ectodomain	−	Sekiya et al. (2003)
Exon 3	c.524A > T	p.Tyr175Phe	Ectodomain, the conserved motif within the active site, invariant tyrosine	−	This study
Exon 3	c.530 T > G	p.Val177Gly	Ectodomain, the conserved motif within active site	−	Kuroiwa et al. (2000)
Exon 4	c.572G > A	p.Arg191Gln	Ectodomain	−	Pras et al. (2012)
Exon 4	c.625C > T	p.Arg209*	Ectodomain	−	Schatz et al. (2010)
Exon 4	c.689_690delCTinsGG	p.Pro230Arg	Ectodomain	−	Wang et al. (2008)
Exon 4	c.710A > C	p.Tyr237Ser	Ectodomain		Sergouniotis et al. (2011)
Exon 4	c.712G > T	p.Gly238Trp	Ectodomain	+	Yamamoto et al. (1999); Gonzalez-Fernandez et al. (1999); Hajali et al. (2009); Iannaccone et al. (2007)
Exon 4	c.718dupG	p.Ala240Glyfs*19	Ectodomain	−	Nakamura et al. (2000)
Exon 4	c.718delG	p.Ala240Profs*7	Ectodomain	−	Makiyama et al. (2014)
Exon 5	c.758 T > G	p.Met253Arg	Ectodomain	−	Ajmal et al. (2012)
Exon 5	c.791 T > G	p.Val264Gly	Ectodomain	+	Hirose et al. (2000)
Exon 5	c.801C > G	p.Cys267Trp	Ectodomain	−	Driessen et al. (2001)
Exon 5	c.824_825delGA	p.Arg275Profs*60	Ectodomain	−	Sergouniotis et al. (2011)
Exon 5	c.832C > T	p.Arg278*	Ectodomain	−	Liu et al. (2015)
Exon 5	c.833G > A	p.Arg278Gln	Ectodomain	−	Pras et al. (2012)
Exon 5	c.839G > A	p.Arg280His	Ectodomain	+	Gonzalez-Fernandez et al. (1999); Nakamura et al. (2000); Kuroiwa et al. (2000); Sato et al. (2004)
Exon 5	c.841 T > C	p.Tyr281His	Ectodomain	−	Nakamura et al. (2000); Nakamura and Miyake (2002)
Exon 5	c.880G > C	p.Ala294Pro	C-terminal transmembrane domain	+	Gonzalez-Fernandez et al. (1999); Schatz et al. (2010)
Exon 5	c.913_917delGTGCT	p.Val305Hisfs*29	C-terminal transmembrane domain	−	Ajmal et al. (2012)
Exon 5	c.928delCinsGAAG	p.Leu310GluVal	C-terminal transmembrane domain	+	Nakamura et al. (2000); Nakamura and Miyake (2002); Sato et al. (2004); Wang et al. (2008); Liu et al. (2014); Makiyama et al. (2014)
Exon 5	c.955 T > C	p.*319Argext*32	C-terminal cytosolic tail	−	Sergouniotis et al. (2011)

^a^‘+’ indicates that the mutants were constructed for this mutation and the analysis of biochemical defects was performed (Yamamoto et al. 1999; Cideciyan et al. 2000; Lidén et al. 2001); ‘–’ indicates that the mutants analysis have not been reported

High variability of the disease’s phenotype is observed among patients with FA carrying *RDH5* mutations. They show a variable visual acuity and variation in the density of white flecks (from minimal white dots or even normal fundus to numerous larger coalescent spots) (Sergouniotis et al. 2011; Ajmal et al. 2012). Despite the observed phenotypic variability, the presence of white dots appeared to be a common feature in patients with FA. These retinal flecks are hypothesised to be the effect of an accumulation of toxic retinyl esters in the RPE as the result of 11-*cis* retinol dehydrogenase disruption (Driessen et al. 2000). However, it is known that, with increasing age in the patients with FA or after uveitis, the dots may fade and become smaller and discrete, especially in the far periphery of the fundus (Yamamoto et al. 2003; Imaizumi et al. 2005; Sergouniotis et al. 2011). Patients with mutations in the *RDH5* gene can manifest a non-progressive or progressive form of the disease. It has been reported that individuals with or without cone dystrophy also presented varying degrees of severity of FA (Nakamura et al. 2000, 2003; Sergouniotis et al. 2011; Ajmal et al. 2012). Moreover, different phenotypes have been observed in patients with the same mutation, for example, c.928delCinsGAAG (p.Leu310GluVal), which is the most commonly identified *RDH5* gene mutation (Nakamura et al. 2000, 2003; Nakamura and Miyake 2002; Sato et al. 2004; Pras et al. 2012; Ajmal et al. 2012).

Therefore, based on the complete review of the literature, it is difficult to establish any valid correlation between the *RDH5* variants and the disease. There is no significant association between the localisation or the type of *RDH5* mutation with the severity of the disease phenotype (including electrophysiological observations or the presence/absence of cone dystrophy) (Sato et al. 2004; Niwa et al. 2005; Sergouniotis et al. 2011; Pras et al. 2012).

## Discussion

The differential diagnosis of flecked retina/white dots syndromes can be difficult using routine ophthalmological examination. In cases of FA with progressive cone dystrophy, signs and symptoms may be non-specific and lead to misdiagnosis. Small white-yellow retinal lesions could indicate the diagnosis of fundus flavimaculatus, familial dominant drusen or retinitis punctata albescens (De Laey 1993; Walia et al. 2009). Moreover, phenotypic variability in the fundus appearance of patients with FA has been described (Sergouniotis et al. 2011; Ajmal et al. 2012). Electrophysiological findings, together with the appropriate genetic analysis, appear to be crucial tools in the differential diagnosis of FA (Pras et al. 2012). Although decreased scotopic ERG responses could appear in many different conditions (retinitis punctata albescens, FA, FA with progressive cone dystrophy and Stargardt disease), their normalisation after 120 min of dark adaptation is observed mostly in FA (Table 2) (Yamamoto et al. 1999; Kanski 2003). However, retinitis punctata albescens due to *RLBP1* mutation (Bothnia dystrophy) may be more difficult to distinguish, as in the early stages, there is phenotypic overlap with FA. Some patients with Bothnia dystrophy show a dramatic improvement in electroretinograms after prolonged dark adaptation (Burstedt et al. 2008; Gränse et al. 2001), while some patients with FA may present a minimal change, even after several hours of dark adaptation (Sergouniotis et al. 2011).

**Table 2:** Comparison of conditions with the symptom of small white-yellow retinal lesions

	Fundus albipunctatus	Fundus albipunctatus with progressive cone dystrophy	Retinitis punctata albescens	Fundus flavimaculatus (Stargardt disease)	Our patient
Eye fundus	Numerous small white-yellow retinal lesions	Numerous small white-yellow retinal lesions	Numerous small white-yellow retinal lesions	Numerous fleck-like yellow retinal lesions	Numerous small white-yellow retinal lesions
Visual field	Normal	Can be constricted	Constricted	Can be constricted	Peripherally constricted
Retinal vessels	Normal	Normal	Attenuated	Can be attenuated	Normal
ERG	Depressed rods responses	Depressed rods responses	Depressed rods responses	Variable	Depressed rods responses
ERG after prolonged dark adaptation	Proper scotopic responses	Proper scotopic responses	Depressed scotopic response	Variable	Proper scotopic responses
mfERG	Normal	Reduced cones density	Normal	Decreased central	Reduced cones density in peripheral rings

Optimistically, recent studies provide hope for the successful treatment of patients diagnosed with FA. Studies on mouse models of FA demonstrated a significant improvement in rod and cone visual function after treatment with 9-*cis* retinal (Maeda et al. 2006). Moreover, the latest pilot clinical testing on a group of patients with FA revealed that treatment with 9-*cis*-β-carotene as a food supplement led to a considerable visual improvement. It is very promising, as there has been no reported treatment resulting in a significant improvement in the visual functions in patients with retinal dystrophy to date and, what is more, this approach will also be helpful for some patients with retinitis pigmentosa (Rotenstreich et al. 2010, 2013).

Genetic analysis of the *RDH5* gene (exons 2–5) in our patient revealed a novel, homozygous mutation c.524A > T in exon 3. The change of the chemical properties of the substituted amino acids and the mutation’s predicted influence on the protein function indicate that the p.Tyr175Phe mutation is probably pathogenic. Moreover, tyrosine at position 175 of the *RDH5* protein is localised within the active site of the enzyme, and was described as invariant tyrosine (Simon et al. 1996). It is known that invariant tyrosines are found in all short-chain alcohol dehydrogenases. The Tyr-X-X-X-Lys sequence motif, a part of the substrate binding (active) site, is the most conserved element in SDRs (Persson et al. 1991; Jörnvall et al. 1995). To date, only one mutation in this highly conserved motif of the human *RDH5* enzyme (Tyr-Cys-Val-Ser-Lys) has been identified. It was a substitution of valine to glycine at amino acid position 177. This variant was found in a boy with FA, who was a compound heterozygote of p.Val177Gly and p.Arg280His (Kuroiwa et al. 2000), but also in a boy diagnosed with familial fleck retina with night blindness (a heterozygote of p.Val177Gly and p.Leu310GluVal) (Hayashi et al. 2006).

The invariant tyrosine, together with the lysine at position 179 (of the human *RDH5*) within the conserved motif and serine-163 (shown in Fig. 1), are involved in the catalytic mechanism (as putative active site residues), but only tyrosine located within this sequence is rigidly conserved in the SDR superfamily (Jörnvall et al. 1995; Filling et al. 2001; Oppermann et al. 2003). The role of the invariant tyrosine was analysed in the most studied member of the SDR superfamily: Drosophila alcohol dehydrogenase (ADH). Albalat and González-Duarte (1992) constructed a Drosophila alcohol dehydrogenase, in which the invariant tyrosine (at amino acid position 152) was substituted by phenylalanine. Drosophila alcohol dehydrogenase-phenylalanine-152 revealed no enzymatic activity. Therefore, it is very likely that substitution of the invariant tyrosine to phenylalanine in human retinol dehydrogenase (*RDH5*) protein would have a similarly damaging effect to that reported in Drosophila ADH.

The segregation analysis of the presence of the c.524A > T mutation in the family studied was found to be consistent with the autosomal recessive mode of inheritance. It revealed that both the proband’s parents are heterozygous carriers of this novel substitution. Therefore, it is highly probable that they are related. We did not confirm this assumption based on the exact pedigree data, but the parents’ families come from villages located in very close proximity.

To conclude, we have presented a brief but complete review of the literature on FA, focusing on the genetic background of the disease. Our study expands the spectrum of *RDH5* mutations, as we also report the novel mutation in the 11-*cis* retinol dehydrogenase 5 gene. This study is the first report of a *RDH5* gene mutation that affects the invariant tyrosine, one of the most conserved amino acid residues in SDRs, crucial for these enzymes’ activity. The location of the substitution, together with the mutation’s predicted influence on protein function, indicate that the p.Tyr175Phe mutation is probably pathogenic and can be recognised as the cause of FA. Moreover, we have presented the first molecular evidence for 11-*cis* retinol dehydrogenase 5 (*RDH5*) gene mutation in a Polish patient with this rare retinal disease. This study may also help clinicians to improve the difficult process of FA differential diagnosis, in which genetic analysis is an indispensable element, which would enable the correct treatment of patients.
